# Indexing of Left Atrial Volume by Body Surface Area and Height in a Brazilian Population without Previous Heart Disease and with a Normal Heart on Echocardiography. Behavior in Obese and Overweight Patients

**DOI:** 10.26502/fccm.92920304

**Published:** 2023-01-29

**Authors:** Edmundo J. Nassri Camara, Flávia R. do Prado Valladares, Ng Kin Key, Paloma Fonseca Santana, Jun Ramos Kawaoka, Thais Harada Campos, Marcus Ribeiro de O. Santana, Alex Costa Cunha, Danilo Sousa Sampaio, Gustavo Pinheiro Santana, Luis Gustavo S. Brito, Narjara de O. Cardoso Dourado, Saulo Jende Nascimento, Alice Povoa A. L. Lira, Naily N. do Nascimento, Romeu Pacheco F. dos Santos, Sérgio Rodrigo F. Rocha, Thaise Gordiano Machado

**Affiliations:** 1 Federal University of Bahia, Brazil; 2 Hospital Ana Nery, Salvador – Bahia, Brasil

**Keywords:** Cardiac Disease, Echocardiograms, Increased Left Atrial

## Abstract

**Background:**

Left atrial (LA) volume indexing for body surface area (BSA) may underestimate LA size in obese and overweight people. Since LA volume is a risk marker for some cardiovascular events, it is suggested that indexing for height would be an alternative more appropriate method. The aims of this study were to find normal and the best cutoff values for LA volume indexed for height in our population.

**Methods:**

Echocardiograms from 2018 to 2021 were reviewed and patients without known cardiac disease and completely normal echocardiograms that had the left atrial volume (LAvol) measured by biplane Simpson’s method were included. LAvol was indexed by BSA (ml/m^2^), by height (LAvol/m), by height raised to exponent 2.7 (ml/ m^2.7^) and by height squared (ml/h^2^).

**Results:**

A total of 545 patients, 50.5 ± 13.4 y., 335 females (61,5%) were analyzed. There were 145 normal weight (26.6%), 215 overweight (39.4%), 154 obese (28.3%) and 31 low weight (5.7%) patients. To establish normal values we included only the normal weight group and considered normal values from 2SD below to 2SD above the mean. Mean and normal values were: LAvol/h 26.0 ±4.5, 17 – 35 ml/m, LAvol/ht^2^ 16 ± 2.8, 10.4 - 21.6 ml/ ht^2^ and LAvol/ht^2.7^ 11.4 ± 2.2, 7.0 - 15.8 ml/m^2.7^. The normal LAvol/ht^2.7^ differed between male and female (11.4 ± 2.4 and 12.8 ± 2.6, p < 0.001). LA diameter, LAvol, LAvol/h, LAvol/h^2^ and LAvol/ht^2.7^ increased progressively from low-weight, normal weight, overweight and obese patients (p< 0.0001), but not LAvol/BSA. When indexing LAvol for height, for height^2^ and for height^2.7^ 20.8%, 22.7% and 21.4% of the obese patients, respectively, were reclassified as enlarged LA, and 7.4%, 8.8% and 8.4% of the overweight patients as well. Using ROC curve analysis, LAvol/h^2^ had the highest AUC ant the best predictive value to identify LA enlargement and LAvol/BSA the worst one.

**Conclusions:**

Normal values for LAvol indexed for height by three different methods are described in normal individuals. We reinforce that LAvol indexation for BSA underestimates LA size in obese and overweight patients and in these groups, specially, indexing for height^2^ is probably the best method to evaluate LAvol.

## 1. Introduction

Increased left atrial (LA) volume is a risk marker for atrial fibrillation, stroke, and heart failure, and it has been correlated with increased cardiovascular mortality [1–7]. International guidelines and clinical practice recommend that LA volume be indexed to body surface (B.S.A.) [8,9]. However, in obese individuals there is a tendency to underestimate these values, often placing individuals with an enlarged left atrium as normal, after indexing by a high B.S.A.. This is an important problem, because obesity is associated with atrial fibrillation [10] and it appears to be mediated by left atrial dilatation [11]. The prevalence of obesity is increasing, it is likely that prevalence of obesity- related AF will increase as well. B.S.A. is an isometric measure, which assumes that LA size has a linear relationship to body size. However, organ size (i.e., heart size) and body size do not grow proportionally; in other words, their relationship is nonlinear [12]. Many parameters can be indexed for height, especially in obese individuals. Alternative forms of scaling, including allometric size variable such as height raised to an exponent, may help address this issue [12,13]. The normal values and how the left atrial volume indexed for height behaves in obese and overweight individuals under normal conditions of cardiovascular health is not well explained in the literature, especially in a Brazilian population. The current work aims to clarify these questions.

## 2. Methods

This is a pilot, observational, descriptive, retrospective study. Echocardiographic data, age, sex, weight, height and body surface were obtained from the data file of the echocardiography service of Hospital Ana Nery, from 2018-2021. Patients with normal echocardiograms, in sinus rhythm, with no history or evident cardiovascular disease (mild systemic arterial hypertension and non-insulin dependent diabetes without target organs involvement), were included independent of any other factor. In all patients, in addition to LA volume, LA and LV dimensions, wall thickness, left ventricular mass, LV ejection fraction, and aortic root diameter were also recorded. Left atrial volumes were obtained by the Simpson’s biplane method of disks using apical four chamber and two chamber views at ventricular end-systole. The data of each patient were reviewed to verify there was no missing data and to be sure that the exam was normal, with special attention to LV diastolic dysfunction parameters, such as the E’ waves of the medial and lateral mitral annulus. Exclusion criteria: any parameter of abnormality such as LA maximum diameter at the parasternal long axis > 40mm, indexed LA volume > 34ml/m^2^, left ventricular wall relative thickness > 0.42, increased left ventricular indexed mass, ejection fraction less than 55%, any change suggesting LV diastolic dysfunction such as reduced medial (< 7cm/s) or lateral (<10cm/s) and mitral regurgitation more than minimal. A total of 645 charts reported as normal echocardiograms were reviewed, and 545 patients were included in the study. The protocol of the study was approved by the Research Ethics Committee of the Hospital Ana Nery (HAN/SESAB), Salvador-BA, which waived the need to obtain written informed consent.

The LA volume were than indexed for B.S.A., height, height^2^ and height^2.7^ in four groups of patients:

- Group I, low weight (B.M.I. < 20 kg/m^2^)
- Group II, normal weight (B.M.I. ≥ 20 kg/m^2^ and < 25 kg/m^2^),
- Group III, overweight (B.M.I. ≥ 25 kg/m^2^ and < 30 kg/m^2^),
- Group IV, obese (B.M.I. ≥ 30 kg/m^2^).

## 3. Data Analysis

Quantitative data are described as mean ± standard deviation and IC 95%. Categorical data are described as an absolute number and percentage. The means, standard deviations (SD) and 95% confidence intervals (CI) of the quantitative variables were obtained for the total of patients and for the subgroups. To comparisons, we considered normal indexed left atrial volume for height, height^2^ and height^2.7^ the interval from 2 SD below and 2 SD above the mean of the normal weight group (B.M.I. ≥ 20 kg/m^2^ and < 25 kg/m^2^). Student’s t test was used to compare means between two independent groups, and the ANOVA test of variance for comparisons between the three groups according to B.M.I. (normal, overweight, and obese) with the Bonferroni test for post hoc analysis between pairs of groups. The chi-square test was used to compare categorical variables. Receiver operating characteristic (ROC) curve analysis was used to identify the indexation method that best predicted increased LA. Statistical analysis were performed with IBM SPSS statistics version 21. Statistical significance was considered as P < 0.05 two-tailed.

## 4. Results

**Figure 1:**
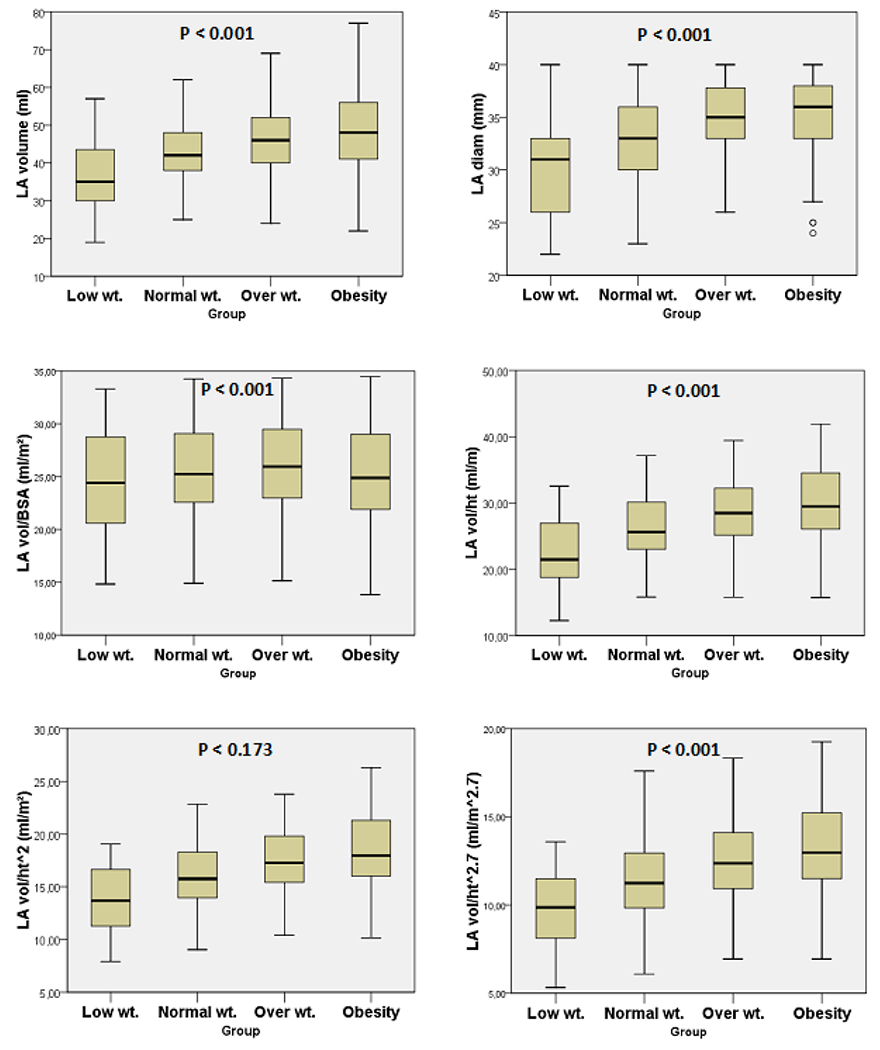
Left atrial volume indexed by B.S.A. and by height (using three different methods). Mean ± SD.

**Figure 2:**
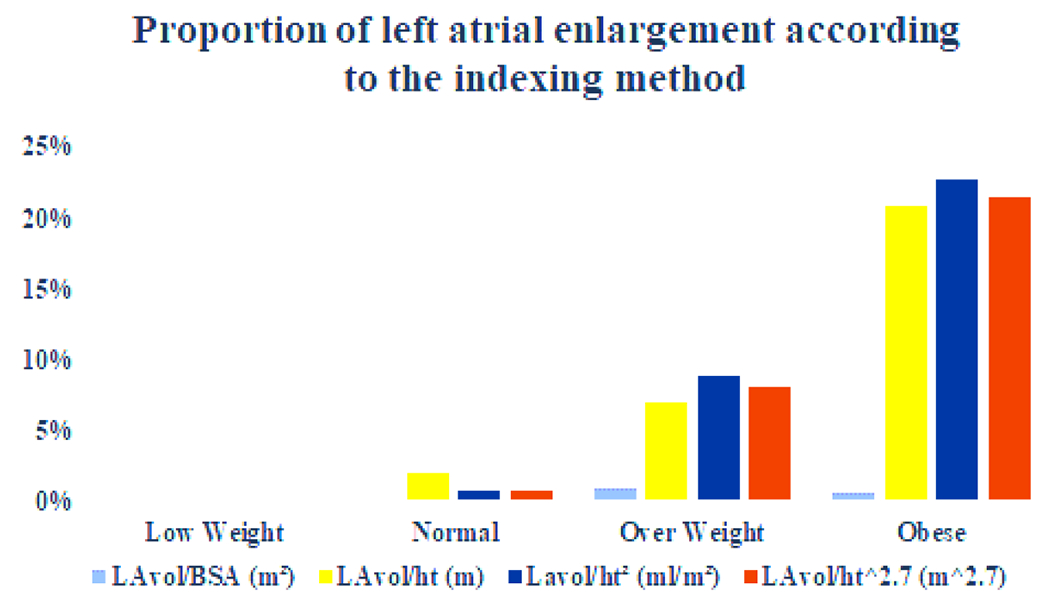
Proportion of left atrial enlargemente according to the indexing method.

**Figure 3:**
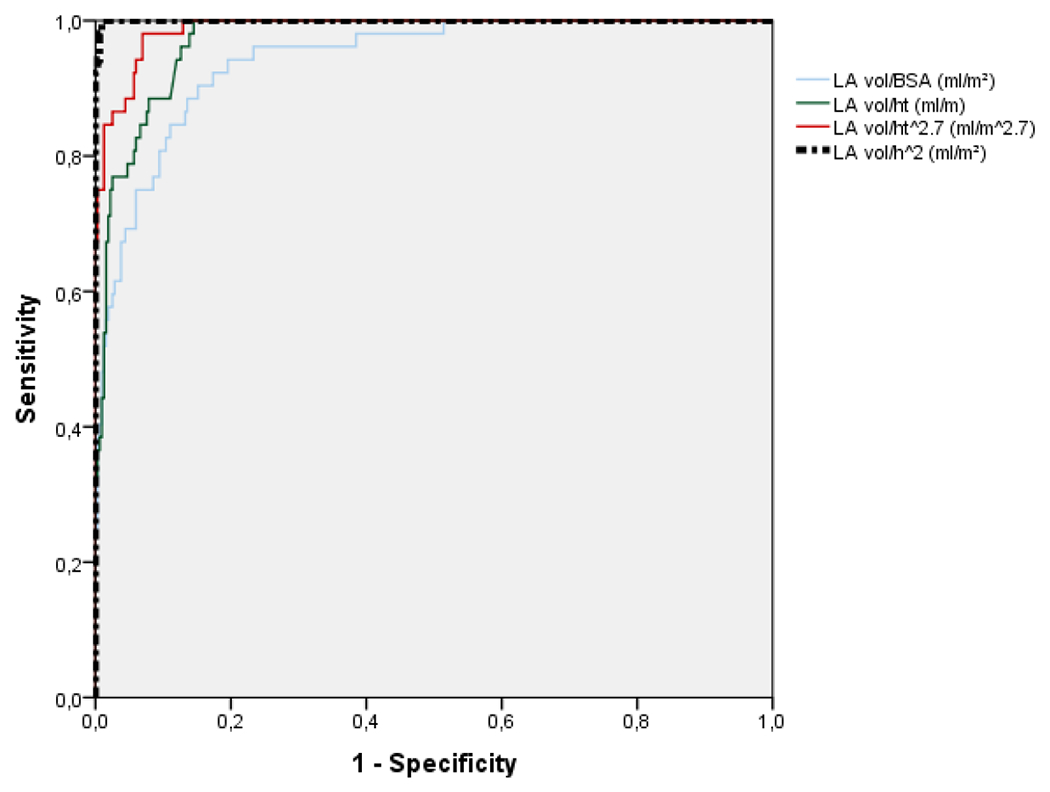
Receiver operating characteristic (ROC) curve analysis for prediction of enlarged LA in obese or overweight patients. Enlarged LA was defined as at least two abnormal of the three methods of volume indexation by height. BSA, body surface area; ht, height; m^2.7, meters raised to exponent 2.7; m^2, meters squared.

A total of 545 patients, mean age 50.5 ± 13.4 y., 335 females (61,5%) and 210 males (38.5%) were analyzed. The anthropometric measurements are detailed in table I. There were 145 normal weight (26.6%), 215 overweight (39.4%), 154 obese (28.3%) and 31 low weight (5.7%) patients. Obesity was more commonly seem in females (75%) just as there was a trend towards more overweight in women (59%). The LA diameter and volume progressively increased from low-weight (Low-wt), normal, overweight (Over-wt) and obese groups (table II and figure 1). When indexed by BSA the LA volume did not differ between groups. Otherwise, when indexing by height, height^2^ and height^2.7^, we observed a progressive and statistically significant increase in the volume of the left atrium (figure 1). The normal values of LA volume according to height, height^2.7^ and height^2^ (allometric measurements) were obtained by the mean ± 2 standart deviations (SD) in the normal weight group. For height the normal values varied from 17.0 to 35.0 ml/m (mean ± SD 26.0 ±4.5). For height^2^ the normal values of LA volume varied from 10.4 to 21.6 (mean ± SD 16.0 ± 2.8). For height^2.7^ the normal values varied from 7.0 to 15.8 ml/m^2.7^ (mean ± SD 11.4 ± 2.2). The normal LA volume for height^2.7^ differed between male and female (11.4 ± 2.4 and 12.8 ± 2.6, p < 0.001). Table III describes the normal values and the maximum limit of normality for indexing the left atrium by height, height^2^ and height^2.7^. Sixteen of 215 (7.4%) of overweight patients and 32 of 154 (20.8%) obese patients were reclassified from normal to dilated left atrium when indexed by height, 19 of 215 (8.8%) and 35 of 154 (22.7%), respectively, when indexed by height^2^, and 18 of 215 (8.4%) and 33 of 154 (21.4%), respectively, when indexed by height^2.7^ according to gender (figure 2). In the normal weight group, only one patient was reclassified as enlarged LA when indexed by height^2^ or by height^2.7^ and 3 patients were reclassified when indexed by height. No low-weight patients were reclassified as enlarged LA when indexed by height, using any one of the three methods. The results of ROC analyses for the best cutoff values to identify LA enlargement in obese or overweight patients are shown in Figure 3 and Table IV. LA was considered enlarged if at least two of the three indexation methods by height were above normal values. LA volume indexation by height^2^ had the highest area under curve (AUC 1.0, 95% CI 0.999-1.0) followed by LAvol/ht^2.7^ (AUC 0.989, 95% CI 0.98-0.997), LAvol/h (AUC 0.972, 95% CI 0.957-0.987) and LAvol/BSA (AUC 0.943, 95% CI 0.914-0.973). Operational cutoff values with corresponding predictive characteristics for obese or overweight patients are presented in Table IV. Interestingly, we found 29.2 ml/m^2^ the best cutoff value if we use LAvol/BSA to the diagnosis of LA enlargement in obese or overweight patients.

## 5. Discussion

Our study shows the normality for left atrial volume when indexing for height instead of indexing for body surface area. We used three different methods: volume/height (meters), volume/m^2^ (meters squared) and volume/height^2.7^ (meters raised to exponent 2.7), the last two are allometric measures. The LA volume indexed by BSA did not differ between groups and in obese patients it was even slightly smaller than in normal or overweight patients. When indexed by any of the three methods using height, LA volume increased progressively and linearly from the underweight, normal, overweight, and obese groups. A higher prevalence of atrial fibrillation and heart failure with preserved ejection fraction is recognized in obese patients, and it may be mediated by left atrial enlargement [10,11,14]. However, indexing LA size in obese or overweight patients is a challenging, and there are important limitations [9]. In this study, we found that 21 to 23% of obese patients and 7 to 9% of overweight patients with normal LA size when indexed by the BSA already had LA enlargement when indexed by height, using any of the three methods. It has been recognized that indexing many echocardiographic parameters for body size in obese patients is a challenging. Singh et al. recommend using height-based indexing instead of BSA in obesity [9]. They proposed the LA volume to be indexed for height^2^ and using the cutoffs proposed by the The European Society of Cardiology (ESC). The ESC guidelines on management of arterial hypertension in adults recommend using LA volume indexed to height^2^ to define normal LA size (≤18.5 mL/m^2^ for men; ≤16.5 mL/m^2^ for women) [15]. In our population we found different cutoffs for normality of LA volume by height^2^ (maximum limit 21.6ml/m^2^), and it was not different for men or women. Applying the limits proposed by the ESC, we would make the diagnosis of dilated LA in 53% of our overweight patients and in 62% of the obese patients, and even in 36% of eutrophic patients and normal heart on echocardiography. So, it doesn’t seem like we should use these cutoffs in our population, and we suggest to review this limit in other populations as well. However, an important point of our work is that we uniformly measured LA volume by Simpson’s biplane method, which is recommended by echocardiography societies, while the method used for that ESC recommendation was the ellipsoid, which may explain differences. All three methods of height indexing for LA volume proved to be excellent predictors of enlarged LA in obese or overweight patients, with height squared indexing (LAvol/h^2^) being the strongest of them. Interestingly, we found 29.2 ml/m^2^ the best cutoff value if we use LAvol/BSA to the diagnosis of LA enlargement in obese or overweight patients instead of 34ml/m^2^ as universally used. In this work we show normal values of left atrial volume according to height using three different methods in eutrophic patients (normal weight, normal BMI) and it is suggested to improve LA size classification in obese and overweight patients. It seems likely that it can be used as well, without prejudice, across the entire weight and body mass index range. We have some limitations in this work. Initially, this is a single-center, retrospective study. It is still necessary to validate these data in other populations and with a larger sample. It was not possible to verify inter and intra observer variability. It was not possible to correlate these data with survival or clinical and cardiovascular events. We are already investigating these findings in pathological conditions, to observe the impact of reclassify the LA size and the severity of dilatation in obese and overweight patients.

## Supplementary Material

1

## Figures and Tables

**Table I:** Anthropometric data on the total number of patients and in the normal weight, underweight, overweight, and obese groups.

	Total			Low-wt	Normal	Over-wt	Obese	
		Min	Max					P
Age	50.5 13.4)	17	83	45.4 17.5)	51.6 (14.4)	51.1 12.7)	49.8 12.2)	0.096
Weight (Kg)	73.4 (15.7)	27	139	49 (8.2)	62 (7.7)	73.4 (8.8)	89.1 14.3)	<0.001
Height (cm)	163.1 (9.4)	135	192	164.5 (9.1)	163.5 (9.2)	163.6 9.6)	161.8 9.3)	0.214
B.S.A. (m^2^)	1.79 (0.21)	1.1	2.5	1.52 0.17)	1.67 (0.15)	1.80 (0.16)	1.93 0.19)	<0.001
m^2.7^	3.78 (0.59)	2.25	5.82	3.9 (0.57)	3.8 (0.59)	3.8 (0.61)	3.7 (0.57)	0.224
m^2^	2.67 (0.31)	1.82	3.69	2.71 0.30)	2.68 (0.31)	2.68 (0.32)	2.63 (0.30)	0.220
B.M.I. Kg/m^2^)	27.6 (5.3)	11.2	53.4	18.0 (1.8)	23.1 (1.3)	27.4 (1.3)	33.95 3.9)	<0.001

**Table II:** Left atrium sizing and indexing by body surface area between normal weight, low weight, overweight and obese patients.

	Total	Low-wt	Normal	Over-wt	Obese	P
LA diam (mm)	34.2 (3.7)	30.4 (4.8)*	33.0 (3.6)	34.9 (3.2)*	35.3 (3.3)*	<0.001
LAvol (ml)	45.5 (9.3)	37.4 (9.6)^a^	42.6 (7.9)	46.6 (8.6)*	48.3 (9.8)*	<0.001
LAvol/B.S.A.	25.5 (4.5)	24.6 (5.2)	25.6 (4.3)	26.0 (4.4)	25.1 (4.8)	0.173
LAvol/ht	27.9 (5.4)	22.7 (5.3)*	26.0 (4.5)	28.5 (4.8)*	29.9 (5.7)*^x^	<0.001
LAvol/ht^2.7^	12.2 (2.6)	9.8 (2.2)*	11.4 (2.2)	12.4 (2.3)*	13.3 (2.8)*+	<0.001
LAvol/ht^2^	17.1 (3.4)	13.8 (3.1)*	16.0 (2.8)	17.4 (3.0)*	18.5 (3.6)*+	<0.001

* P<0.01 comparing to normal weight;

a P<0.02 versus normal weight;

+ P<0.01 obese versus overweight;

x P=0.055 obese versus overweight

**Table III:** Normal values and the maximum normal for left atrium indexing by height, height^2^ and height^2.7^.

	Normal	Maximum normal

LAvol/B.S.A.	25.6 ± 4.3	34.2

LAvol/ht (ml/m)	26.0 ±4.5	35.0

LAvol/ht^2^ (ml/ m^2^)	16 ± 2.8	21.6

LAvol/ht^2.7^ (ml/ m^2.7^)	11.4 ± 2.2	15.8
male	11.4 ± 2.4	16.2
female	12.8 ± 2.6	118.0

**Table IV:** Results of ROC analyses to identify LA enlargement* in obese or overweight patients.

Variables			Optimal cutoff value		
	AUC	95% CI		Sensitivity	Specificity
LAvol/BSA (ml/m^2^)	,943	0.914-0.973	29.2	90.40%	84.90%
LAvol/ht (ml/m)	,972	0.957-0.987	34.4	84.60%	93.40%
LAvol/ht^2.7 (ml/m^2.7^)	,989	0.980-0.997	15	98.10%	93.10%
LAvol/ht^2 (ml/ht^2^)	1,000	0.999-1.000	21.5	100%	99.10%

* LA enlargement was defined if at least two of the three indexation methods by height were above normal values.
