# Supplementary material for: Indexing of Left Atrial Volume by Body Surface Area and Height in a Brazilian Population without Previous Heart Disease and with a Normal Heart on Echocardiography. Behavior in Obese and Overweight Patients
Source: Cardiol Cardiovasc Med. Author manuscript; Available in PMC 2023 Mar 16. (PMC10019596; doi:10.26502/fccm.92920304)
Supplement: 1 [file NIHMS1875753-supplement-1.pdf]

	N	Normal	Maximum normal	P
LAvol/B.S.A.		25.6 ± 4.3	34.2	
male	69	25.4 ± 4.4	34.2	0.73
female	76	25.7 ± 4.3	34.3	
LAvol/ht (ml/m)		26.0 ±4.5	35.0	
male	69	26.4 ± 4.6	35.6	0.32
female	76	25.7 ± 4.4	34.5	
LAvol/ht² (ml/ m²)		16 ± 2.8	21.6	
male	69	15.6 ± 2.9	21.4	0.175
female	76	16.3 ± 2.8	21.9	
LAvol/ht$^{2.7}$ (ml/ m$^{2.7}$)		11.4 ± 2.2	15.8	
male	69	11.4 ± 2.4	16.2	0.005
female	76	12.8 ± 2.6	18.0	

Supplementary Table I: Normal values and the maximum normal (mean + 2 SD) for left atrium indexing by height, height² and height$^{2.7}$ and by sex.
